# Research on Influencing Factors and Dimensions of Health Literacy in Different Age Groups: Before and After the COVID-19 Era in Chongqing, China

**DOI:** 10.3389/fpubh.2021.690525

**Published:** 2021-09-06

**Authors:** Peiying Yang, Yanran Ou, Hailin Yang, Xuyan Pei, Jiarui Li, Yuxing Wang, Fang Tan, Xin Zhao, Weiwei Liu

**Affiliations:** ^1^School of Public Health and Management, Chongqing Medical University, Chongqing, China; ^2^Department of Public Health, Our Lady of Fatima University, Valenzuela, Philippines; ^3^Chongqing Collaborative Innovation Center for Functional Food, Chongqing University of Education, Chongqing, China

**Keywords:** health literacy, different age groups, influencing factors, health literacy dimensions, COVID-19, Chongqing

## Abstract

**Background:** Understanding the levels of health literacy among different groups is essential for better public health interventions targeting specific subgroups of the population. Additionally, this article explores the prevalence and influencing factors of the health literacy levels of different age groups during the COVID-19 epidemic.

**Methods:** Multistage stratified cluster random sampling and the Probability Proportion to Size (PPS) method were used to select permanent residents aged 15–69 in Chongqing (54,706) for the questionnaire survey. The survey period is from July 2019 and July 2020. Single-factor analysis and logistic regression models were used to study the relationship between demographics, socioeconomic factors, other independent covariates, and health literacy.

**Results:** The health literacy levels of residents declined with age, and there were significant differences in health literacy levels between age groups (χ^2^ = 3332.884, *P* < 0.05). As far as the factors affecting health literacy level are concerned, high education and high income are the protective factors for health literacy level for residents of all ages. For adolescents (OR = 1.383, 95% CI: 1.217–1.571), young adults (OR = 1.232, 95% CI = 1.117–1.358), and middle-aged people (OR = 1.096, 95% CI = 1.017–1.182), residence in rural areas was a protective factor. In terms of the dimensions of health literacy, in particular, elderly health literacy in 2020 in Scientific Health Concepts, Safety and First Aid, Basic Medical Care decreased significantly compared with 2019.

**Conclusions:** For adolescents, young adults, middle-aged people, to solve the problem of urban and rural health quality gap, we should not only use the geographical division, but also consider the social population and socio-economic differences. For the elderly, the following four dimensions of health literacy need to be paid more attention than those of other age: Basic Knowledge and Concepts, Scientific Health Concepts, Safety and First Aid, and Basic Medical Care. A lack of knowledge on the prevention and treatment of chronic diseases is the main reason for the recent decline in health literacy. And the health literacy among residents in major public health emergencies is needed.

## Introduction

In 1998, the WHO defined health literacy as “cognitive and social skills, which determine the motivation and ability of individuals to understand and use information in a way that promotes and maintains good health” (1). A systematic review of existing definitions and models of health literacy proposed the integration of definitions and conceptualizations, that is, the knowledge, motivation, and competencies to access, understand, appraise, and apply health information to make judgments and make decisions in everyday life concerning healthcare, disease prevention and health promotion to maintain or improve the quality of life during the life course (2). According to published studies, health literacy was associated with health outcomes, including physical and mental health, the use of health care services, hospitalization, and mortality (3–6). Factors affecting the level of health literacy included financial deprivation, older age, lower educational level, perceived poor health, poor health status, high use of health care services, low socioeconomic status, male sex, and lack of the ability to effectively utilize Internet information (7, 8). In the past few decades, health literacy has become an important topic in public health research. Despite the increasing amount of attention devoted to health literacy among Chinese health policymakers, researchers, and practitioners, information about the status of health literacy in China, the most populous country in the world, remains scarce.

For China, the general goal of 2030 is as follows: by 2030, the system of promoting the health of all people will be improved, the development of the health field will be more coordinated, a healthy lifestyle will be popularized, and the quality of health services and the level of health security will continue to improve. In 2019, the National Health and Health Commission showed that the overall level of health literacy of residents in China continued to improve steadily. The level of health literacy has reached 19.17 and 2.11% higher than in 2018, but still at a lower level needed increase. When we focus on local areas, Chongqing performs better in improving residents' health literacy. Chongqing's four major health indicators, “life expectancy per capita, maternal mortality, infant mortality, and under-five mortality,” are all superior to the national average. According to the data of the sixth national census in China in 2020, among the inhabitant in Chongqing, the population aged 15–69 accounted for 75.67%. For adolescents aged 15–24, the results showed that poor health literacy is linked to three psychological disturbances commonly experienced in this age group: perceived stress, depressive symptoms, and impulsivity. For young and middle-aged people aged 30–59 years old, poor health literacy was associated with some adverse health outcomes, such as obesity and smoking, and there were mixed findings about health literacy and medication adherence among those with a chronic illness (9). For elderly people aged 60–69, low health literacy was linked to subjective cognitive decline and morbidity among healthy community-dwelling older adults which should prove useful in the planning of dementia prevention and intervention programs (10). A large number of studies have shown that the levels of adequate health literacy in different age groups is limited (8). There also are a few studies on the health literacy of people of different ages, most of which focus on the study of special populations. It is crucial to study the awareness level of health literacy in different age groups and their respective problems to improve public health interventions in each subgroup of the population.

In addition, in public health emergencies, we can recognize the level of health literacy more intuitively through people's concrete actions. On January 31, 2020, the World Health Organization (WHO) declared that the COVID-19 epidemic was listed as a “Public Health Emergency of International Concern” (PHEIC). When the epidemic occurred, not only were a country's emergency response capabilities and medical standards challenged, but it was also a test of the people's health habits and their lifestyle. The approach to measure the latter is health literacy. Health literacy is very important to prevent individual infectious diseases. In an emergency infectious disease environment, people with low health literacy may not be able to timely obtain effective health knowledge, and implement good health behavior (11). Health literacy is a crucial factor in managing the COVID-19 epidemic and offers a perspective for future studies that target health literacy in the context of virus outbreaks (12). In the face of major public health emergencies caused by new infectious diseases, China has significant advantages in terms of its political system as well as its prevention and control systems, but a lack of national health literacy has become obvious during this epidemic. For example, many people wore masks in the wrong direction and continued to touch their masks with their contaminated hands (13). Although the Internet has been very developed, people do not pay much attention to health knowledge. These results show that people have poor knowledge about health, and it is an underestimated problem not only nationally but also internationally (14).

Therefore, it is of great significance to study the health literacy levels of different age groups before and after the era of COVID-19. In this study, Chongqing health literacy education work has a strong promotion significance in western China. However, there are few researches on the health literacy and its influencing factors of different age groups in Chongqing. Therefore, it is instructive to study the health literacy level of different age groups that reside in this area.

## Methods

### Study Participants and Sampling Procedure

This study belongs to the results of Chongqing area in the National Health Literacy Survey. The study population was residents aged 15–69 years in 39 districts and counties of Chongqing before and after the era of COVID-19, which called permanent residents. Permanent residents involved those who had resided for over 6 months in the past 1 year. However, residents who collectively lived in military bases, hospitals, prisons, nursing homes, dormitories, and other places were excluded. Using streets as urban monitoring points and townships as rural monitoring points. This research was approved by the Ethics Committee of Chongqing Second Normal University, and the research process complied with ethical standards. Obtain written informed consent from each participant or a representative office designated by law.

**Figure 1:**
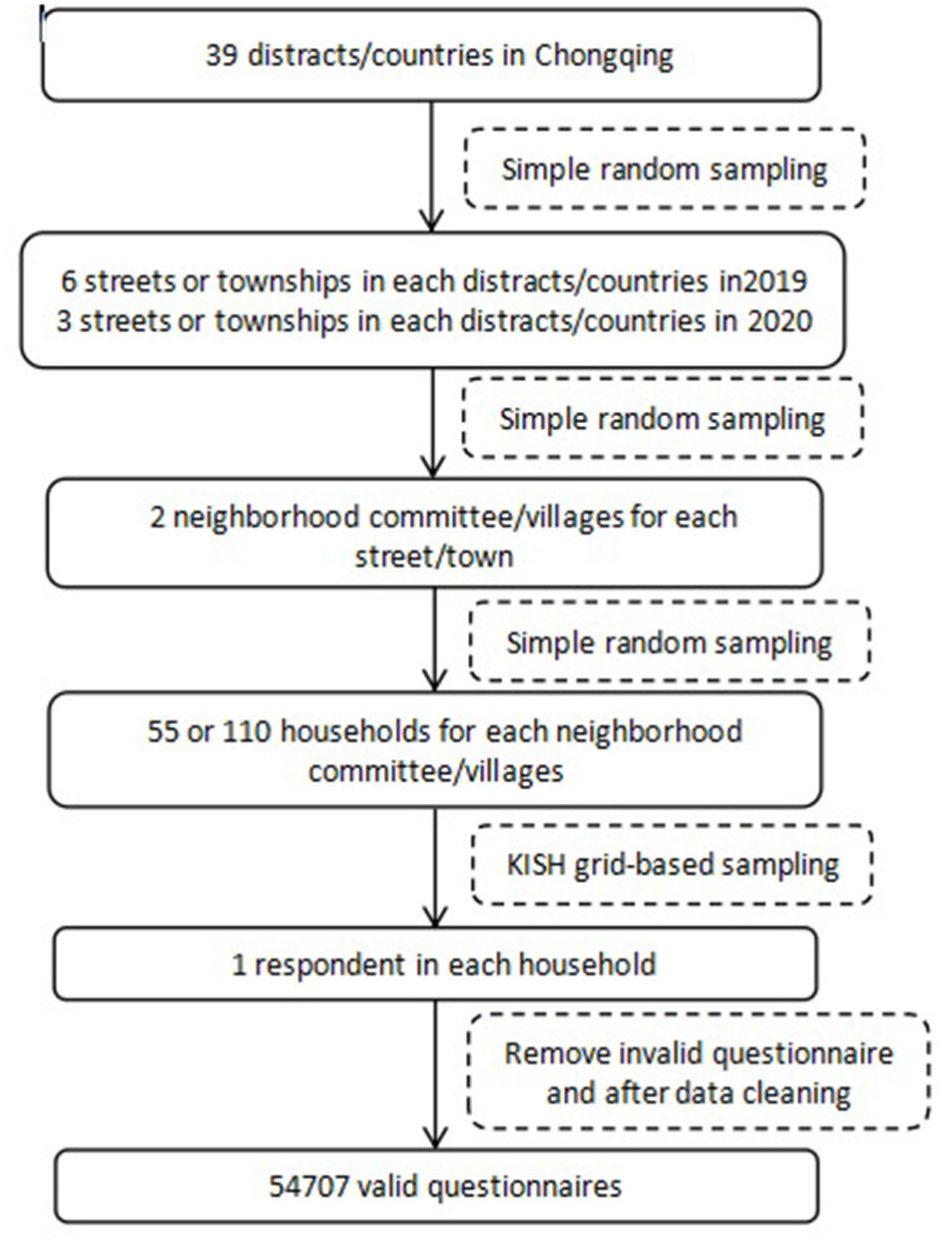
Implementation steps of sampling.

Using multistage stratified cluster random sampling and the PPS method (probability proportional scale sampling), 39 districts and counties in the city were selected as monitoring points. Each district and county selected 6/3 streets/townships (Affected by the epidemic, sampling in 20 years has been halved. 2019 is 6 and 2020 is 3), and 2 neighborhood committees/villages are selected from each street/township. Among them, 55 households were selected from the first 2 villages/neighborhood committees of each street/township. The third neighborhood committee/villages in each street/township makes a list of the households and selects 110 households. One permanent resident aged 15–69 was selected from each household according to the KISH table method for the household survey. Questionnaires with missing values for critical information (address, gender, and age) or health literacy outcome variables were excluded. After data cleaning, 54,706 valid questionnaires were analyzed. See Figure 1 for details.

### Measurements

#### Health Literacy Questionnaire

The Chinese Citizen Health Literacy Questionnaire, which was developed by the China National Center for Health Education, was used in this study. The questionnaire is divided into two parts: first, basic demographic information, such as age, gender, household registration, education level, and occupation. There are 56 questions in the second part which are based on the “Chinese Citizens' Health Literacy-Basic Knowledge and Skills (Trial),” of which 50 questions are included in the calculation of the health literacy points (Cronbach's alpha of was 0.949, and the Spearman–Brown coefficient was 0.984). The second part was further categorized into 3 aspects and 6 problems. The 3 aspects are Basic Knowledge and Concepts (BKC, 22 questions), Healthy Lifestyles and Behaviors (HLB, 16 questions), and Health Skills (HS, 12 questions). The 6 types of problems are Scientific Health Concepts (SHC, 8 questions), Infectious Disease prevention (ID, 6 questions), Chronic Disease prevention (CD, 9 questions), Safety and First Aid (SFA, 10 questions), Basic Medical Care (BMC, 11 questions), and Health Information (HI, 6 questions).

At the scene, a trained investigator equipped with an Android tablet or Android phone with the “Chongqing Health Literacy Monitoring” app entered the home and conducted the questionnaire survey. The questionnaire which asked about the basic demographic characteristics of the residents, 3 aspects and 6 problems.

#### Health Literacy Evaluation

The full score of the questionnaire is 66 points. The total points scored in the 3 aspects BKC, HLB, and HS were 28, 22, and 16, respectively. The total points scored in the 6 problems SHC, ID, CD, SFA, BMC, and HI were 11, 7, 12, 14, 14, and 8, respectively. There were 10 true or false questions, 26 single-choice questions, 16 multiple-choice questions, and 4 situational questions (including 3 single-choice questions and 1 multiple-choice question). True or false and single-choice questions were counted as 1 point for correct answers and 0 points for errors; 2 points were counted for multiple-choice questions that gave the correct answer, and 0 points were counted for wrong choices. Missing value processing: The health literacy monitoring data has not been filled with missing values. For health literacy assessment questions, unanswered questions will be counted as 0 points. Outliers cleaning: according to the actual situation, check the original data, determine the type of error, supplement or correct the data, and remove the questionnaires that are found to be unqualified. The above standards are from the China Health Education Center.

The maximum score of health literacy is 66. $S=\frac{T}{66}\times 100$%, where “S” represents the percentage of the total score of a sample in the total score of the questionnaire and “T” represents the total score of a sample's health literacy. A score for the second part of the questionnaire >80% (80% of 66 points is 53 points) of the total score is regarded as having adequate health literacy, and the percentage of people with adequate health literacy in the total population is the overall level of health literacy. The overall health literacy level of the 3 aspects and 6 problems of health literacy were calculated similarly. In a certain dimension, the number of people who scored more than 80% accounted for the percentage of the total survey population, which is the health literacy level of a certain dimension. $N=\frac{D}{L}\times 100$, where “N” represents the percentage score of a certain dimension of a certain sample in the total score of that aspect/problem of the questionnaire, “D” represents the total score of a certain aspect/problem of a sample's health literacy, and “L” represents the total score of a certain aspect/problem of health literacy. If *N* ≥80, the sample has adequate health literacy in the dimension.

#### Covariates

Age, survey year, place of residence, gender, education level, income, and chronic diseases were all included in the analysis as sociodemographic covariates.

Age was recorded in years and categorized into four age groups for the analyses. The Chinese central government document “Medium and Long-term Youth Development Plan (2016–2025)” referred to youths in the age range of 14–35 years old. Therefore, the international physiological and biochemical indicators were not fully adapted to China. Secondly, the age we set was based on the relevant research on health literacy conducted by the China Health Education Center. The youngest age group of 15- to 29-year-old people, known as “adolescents” because they have a political role, are transitioning into laborers and consumers and starting their own families, thereby increasing their independence from the economy and their emotions (15). People in the second age group are between 30 and 45 years old and are called “young people,” with increasing obligations in terms of family organization, the labor market, and political and civic participation. The study population between the ages of 46 and 59 was classified as “middle-aged adults.” Their status can be defined by complex obligations and stable life plans. Individuals 60 years and older represent senior citizens. Most of them have retired, are facing dwindling opportunities and physical possibilities, and have experienced some serious health management problems (16) (age 15 ~ 29 = 1, 30 ~ 44 = 2, 45 ~ 59 = 3, 60 ~ 69 = 4 groups).

In 2019 and 2020, we monitored the health literacy of Chongqing residents for 2 consecutive years. Gender was classified as “female” or “male” (male = 1, female = 2). According to where people live, they were divided into urban areas and rural areas (rural = 1, urban = 2).

Educational level was assessed using the International Standard Classification of Education (ISCED-97) (17) combined with China's national conditions. Those with only an elementary school education and illiterate residents are less educated. China's compulsory education through junior high school and high school diplomas were regarded as medium educated, and those with a college degree and above were regarded as highly educated (low educational level = 1, medium education level = 2, high education level = 3).

The division is based on the per capita disposable income level in Chongqing (18, 19); residents with a per capita annual income of < ¥3,000 ($434.95) belong to the low-income group; residents with a per capita annual income between ¥3,000 and ¥10,000 ($1449.82) were regarded as the middle-income group; residents ¥10,000 yuan and above were regarded as the high-income group (low-income group = 1, middle-income group = 2, high-income group = 3).

The chronic diseases we defined included hypertension, heart disease, cerebrovascular diseases (such as stroke, cerebral infarction, cerebral thrombosis, etc.), diabetes, malignant tumors, and other chronic diseases (with chronic disease = 1, not suffering from chronic disease = 0).

### Statistical Analyses

All data were double-entered using Microsoft Office Excel 2017, and all data analyses were performed using SPSS 26.0. Descriptive analysis was performed to describe the research population and the level of health literacy. The chi-square test was used to evaluate the relationship between the health literacy possession rate of Chongqing residents and the health literacy level of different dimensions at different ages. Single-factor analysis and logistic regression models were used to study the relationship between demographics, socioeconomic factors, other independent covariates and health literacy. These analyses were performed on the total sample and stratified by age group (15–29 years, 30–44 years, 45–59 years, and above). The level of health literacy was divided into two categories: “no” (a health literacy score <80) and “yes” (a health literacy score ≥80). The criterion of significance was α = 0.05, corresponding to a *P* < 0.05.

## Result

### Single Factors of Health Literacy in Different Age Groups

The health literacy levels of residents declined with age, and there were significant differences in health literacy levels between age groups (χ^2^ = 3332.884, *P* < 0.05). Within the age group of 15–29 years, among which literacy in 2019 was higher than in 2020. The higher the education level and income, the higher the health literacy. People without chronic diseases have higher health literacy.

In the 30 to 44-year-old and 45 to 59-year-old age groups, these five variables were also statistically significant (*P* < 0.05). The results for the other variables were similar to those in the 15 to 29-year-old age group except that the literacy level among those residing in an urban area was significantly higher than among those residing in the countryside.

Within the age group of 60–69, the resulted showed that for elderly residents in Chongqing, the health literacy level in 2019 was higher than that in 2020, those residing in urban areas had higher literacy than those residing in rural areas, and the higher the education level and income level, the higher the health literacy level. See Table 1 for details. Table 1 only selected residents with adequate health literacy.

**Table 1:** Factors associated with health literacy and stratified by age groups—results of Bivariate Analyses.

	**15 **~** 29**	**30 **~** 44**	**45 **~** 59**	**60 **~** 69**
	***n* (%)**	***n* (%)**	***n* (%)**	***n* (%)**
**Survey year**
2019	1,286 (40.2)	2,166 (35.1)	3,096 (20.3)	1,208 (10.9)
2020	760 (37.1)	1,046 (33.0)	1,584 (18.6)	363 (6.9)
χ^2^	4.914	4.150	10.536	64.845
*P*-value	**0.027**	**0.042**	**0.001**	** <0.001**
**Inhabitation**
Urban	843 (39.0)	1,431 (37.2)	1,262 (23.1)	566 (12.8)
Rural	1,203 (39.0)	1,781 (32.4)	3,118 (18.3)	1,005 (8.4)
χ^2^	0.002	22.917	69.421	70.305
*P*-value	0.962	** <0.001**	** <0.001**	** <0.001**
**Educational level**
Low	25 (16.1)	308 (16.6)	1,213 (10.8)	813 (7.1)
Medium	985 (32.3)	1,852 (34.1)	2,966 (25.6)	687 (14.9)
High	1,036 (50.8)	1,052 (51.4)	501 (50.4)	71 (34.5)
χ^2^	210.567	521.288	1389.878	382.358
*P*-value	** <0.001**	** <0.001**	** <0.001**	** <0.001**
**Household per capita income**
Low	249 (32.9)	308 (26.3)	597 (13.3)	333 (7.1)
Medium	743 (37.7)	1,031 (29.6)	1,987 (17.8)	623 (8.7)
High	1,054 (41.8)	1,873 (40.0)	2,096 (25.9)	615 (13.8)
χ^2^	21.548	133.7	341.699	129.724
*P*-value	** <0.001**	** <0.001**	** <0.001**	** <0.001**
**Chronic disease**
No	2,029 (39.2)	3,074 (34.6)	3,831 (20.1)	1,015 (9.8)
Yes	17 (26.2)	138 (30.0)	849 (18.0)	556 (9.3)
χ^2^	4.561	4.159	10.771	0.787
*P*-value	**0.033**	**0.041**	**0.001**	0.375
**Total score possession rate**	2,046 (39.0)	3,212 (34.4)	4,680 (19.7)	1,571 (9.6)

*n (%) refers to the absolute number and percentage of persons having HL (score ≥80). The bold values means significant differences (p < 0.05)*.

### Multiple Factors of Health Literacy in Different Age Groups

Taking health literacy as the dependent variable (Yes = 1; No = 0), we used single-factor analysis of statistically significant survey year, residence, education level, per capita household income, and chronic disease as independent variables in logistic regression analysis. The results showed that for residents of all ages, a high education and high income were protective factors for health literacy level. Compared to 2019, the odds of having adequate health literacy were significantly lower in 2020 across four different age groups. Residence in rural areas was a protective factor for adolescents (OR = 1.383, 95% CI: 1.217–1.571), young adults (OR = 1.232, 95% CI = 1.117–1.358), and middle-aged people (OR = 1.096, 95% CI = 1.017–1.182).” See Table 2 for details.

**Table 2:** Factors associated with health literacy^*^ stratified by age groups-results of the Logistic Regression.

	**15** **~** **29** ^**a**^		**30** **~** **44** ^**b**^		**45** **~** **59** ^**c**^		**60** **~** **69** ^**d**^	
	***OR* (95%CI)**	***P*-value**	***OR* (95%CI)**	***P*-value**	***OR* (95%CI)**	***P*-value**	***OR* (95%CI)**	***P*-value**
**Year**
2019	Ref							
2020	0.842 (0.749–0.947)	**0.004**	0.843 (0.767–0.926)	** <0.001**	0.835 (0.779–1.182)	** <0.001**	0.570 (0.503–0.645)	** <0.001**
**Inhabitation**
Urban	Ref							
Rural	1.383 (1.217–1.571)	** <0.001**	1.232 (1.117–1.358)	** <0.001**	1.096 (1.017–1.182)	**0.017**	0.944 (0.832–1.071)	0.373
**Educational level**
Low	Ref							
Medium	2.576 (1.665–3.986)	** <0.001**	2.578 (2.245–2,961)	** <0.001**	2.701 (2.507–2.911)	** <0.001**	2.122 (1.889–2.383)	** <0.001**
High	5.906 (3.796–9.189)	** <0.001**	5.341 (4.532–6.94)	** <0.001**	7.465 (6.453–8.635)	** <0.001**	5.698 (4.169–7.786)	** <0.001**
**Household per capita income**
Low	Ref							
Medium	1.238 (1.033–1.484)	**0.021**	1.182 (1.015–1.378)	**0.032**	1.327 (1.200–1.468)	** <0.001**	1.224 (1.064–1.408)	**0.005**
High	1.385 (1.157–1.658)	** <0.001**	1.401 (1.204–1.631)	** <0.001**	1.609 (1.447–1.790)	** <0.001**	1.538 (1.317–1.797)	** <0.001**
**Chronic disease**
No	Ref							
Yes	0.669 (0.379–1.183)	0.167	1.007 (0.814–1.246)	0.948	0.951 (0.873–1.036)	0.249	0.935 (0.837–1.044)	0.078

a *Omnibus Tests of model coefficients:χ^2^ = 253.823, P < 0.001; Hosmer-Lemeshow Text: χ^2^ = 17.048, P < 0.05; Predicted percentage correct = 62.4%; Method: Input; n = 5,247, all the respondents were included.*

b *Omnibus Tests of model coefficients: χ^2^ = 585.881, P < 0.001; Hosmer-Lemeshow Text: χ^2^ = 9.106, P > 0.05; Predicted percentage correct = 66.6%; Method: Input; n = 9,336, all the respondents were included.*

c *Omnibus Tests of model coefficients: χ^2^ = 1445.474, P < 0.001; Hosmer-Lemeshow Text: χ^2^ = 4.100, P > 0.05; Predicted percentage correct = 80.5%; Method: Input; n = 2,3781, all the respondents were included.*

d *Omnibus Tests of model coefficients: χ^2^ = 438.791, P < 0.001; Hosmer-Lemeshow Text: χ^2^ = 6.480, P > 0.05; Predicted percentage correct = 90.4%; Method: Input; n = 16,342, all the respondents were included.*

*The bold values means significant differences (p < 0.05).*

* *Measured as score of the “Chinese Citizens' Health Literacy-Basic Knowledge and Skills (Trial)”*.

### Chi-Square Test of Different Dimensions of Health Literacy for Different Age Groups in 2019 and 2020

As shown in Table 3, the year of the survey was significantly correlated with the level of health literacy and the overall health literacy level of each age group declined from 2019 to 2020.

**Table 3:** Factors associated with health literacy in 3 different aspects and 6 types of health problems (2019 and 2020)^*^ stratified by age groups—results of bivariate analyses.

	**15 **~** 29**	**30 **~** 44**	**45 **~** 59**	**60 **~** 69**
	**%**	**%**	**%**	**%**
**Basic knowledge and concepts**
2019	52.6	46.0	28.1	16.4
2020	40.0	37.2	21.3	9.3
χ^2^	79.475	65.876	133.864	146.835
*P*-value	** <0.001**	** <0.001**	** <0.001**	** <0.001**
**Healthy lifestyles and behaviors**
2019	39.5	34.3	22.1	13.4
2020	46.4	40.4	23.9	13.8
χ^2^	24.659	33.614	9.735	0.498
*P*-value	** <0.001**	** <0.001**	**0.002**	0.481
**Health skills**
2019	22.3	20.4	11.9	5.8
2020	36.0	34.2	19.9	8.9
χ^2^	116.972	212.153	278.099	52.700
*P*-value	** <0.001**	** <0.001**	**0.002**	** <0.001**
**Scientific health concepts**
2019	60.9	54.1	39.9	26.8
2020	60.4	54.9	38.2	24.6
χ^2^	0.104	0.602	6.692	9.022
*P*-value	0.747	0.439	** <0.001**	**0.003**
**Prevention and control of infectious diseases**
2019	30.6	27.9	18.0	10.6
2020	43.3	37.6	24.4	13.0
χ^2^	87.738	92.225	136.688	20.170
*P*-value	** <0.001**	** <0.001**	**0.002**	** <0.001**
**Prevention and treatment of chronic diseases**
2019	45.4	37.4	24.2	16.1
2020	27.0	21.2	12.6	5.5
χ^2^	178.807	251.927	461.241	358.142
*P*-value	** <0.001**	** <0.001**	** <0.001**	** <0.001**
**Safety and first aid**
2019	70.0	63.0	44.5	29.3
2020	71.5	64.0	42.5	26.6
χ^2^	1.388	0.987	8.969	13.144
*P*-value	0.239	0.321	**0.003**	** <0.001**
**Basic medical care**
2019	37.4	34.9	24.1	15.3
2020	40.0	36.7	23.9	13.5
χ^2^	3.491	2.872	0.191	9.189
*P*-value	0.062	0.090	0.662	**0.002**
**Health information**
2019	49.5	43.2	27.4	17.0
2020	48.8	45.5	28.3	16.4
χ^2^	0.265	4.331	2.130	0.887
*P*-value	0.607	**0.037**	0.144	0.349

*The bold values means significant differences (p < 0.05).*

* *Measured as score of the “Chinese Citizens' Health Literacy-Basic Knowledge and Skills (Trial)”*.

**Figure 2:**
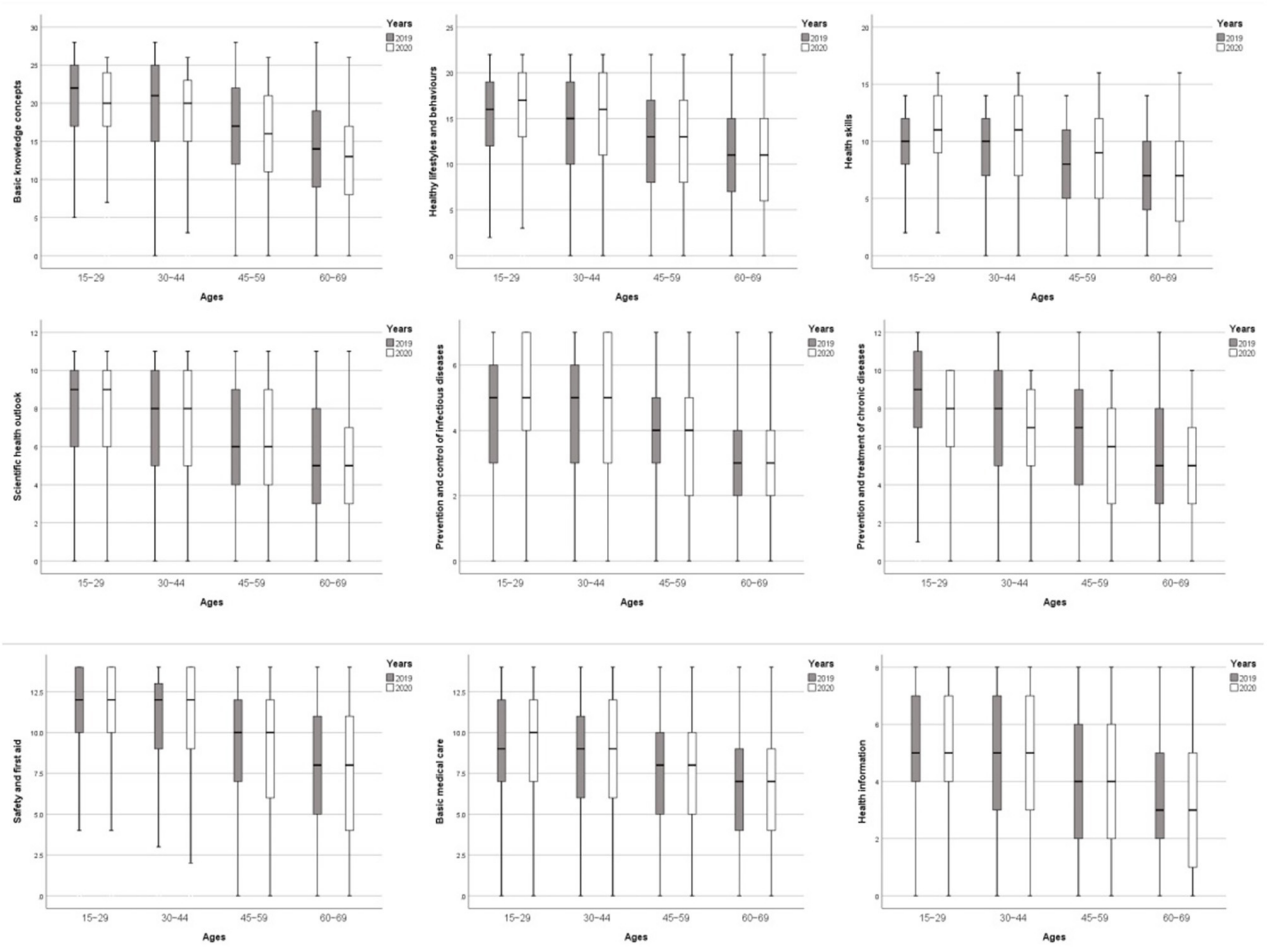
Trends in the scores of the “3 aspects and 6 problems” health literacy at each age.

It can be seen from Table 3 and Figure 2 that among the 3 aspects, “the Basic Knowledge and Concepts” of residents in 2020 were significantly lower than those in 2019 in all age groups, while “Health Skills” were the opposite. In terms of “Healthy Lifestyle and Behavior,” except for the elderly (60–69 years old), the level of health literacy changes was not statistically significant, and for the other age groups, it increased significantly.

From the perspective of 6 problems, only middle-aged people (45–59 years old) and the elderly (60–69 years old) had significantly reduced health literacy levels among the “Scientific Health Concept” and “Safety and First Aid.” In the “Infectious Disease prevention,” the health literacy level of all age groups had increased significantly, while the “Chronic Disease prevention” was the opposite. Only the elderly (60–69 years old) experienced a significant decline in the issue of “Basic Medical Care.” In the “Health Information,” the health literacy level of the entire population and the young and middle-aged population (30–44 years old) had increased significantly. See Table 3 and Figure 2 for details.

## Discussion

For the first time, this article focused on the differences of health literacy in diverse age groups of Chongqing residents before and after the era of COVID-19 and it discussed the influencing factors of various age groups on the level of health literacy in disparate dimensions.

According to the four age groups representing the dissimilar stages in the life course of Chongqing's population, the levels of health literacy were distinct. The health literacy levels of the residents gradually decreased with the natural change in age, which was consistent with previous international studies (15, 20–22). However, we found some new discoveries, and the results showed that in different age groups, under the influence of different factors, there are significant differences in the rate of the residents' health literacy.

### For Adolescent to Middle-Aged People, Sociodemographic Factors Were Important for the Differences in Health Literacy Between Urban and Rural Populations

Our results showed that for the total sample covering all age groups, the health literacy levels of rural residents were significantly lower than those of urban residents, which is consistent with the results of most researchers. However, after conducting a stratified analysis by age and excluding confounding factors, we obtained different results. We found that the health literacy levels of the three age groups of adolescents, young adults, and middle-aged residents living in rural areas were significantly higher than those in urban areas. This meant that regardless of whether there were differences in health literacy between urban and rural populations, living in rural areas cannot solely explain the differences in health literacy between urban and rural areas, and sociodemographic factors must also play an important role (23, 24). Based on the results of this article, to solve the problem of the gap between urban and rural health literacy, we should not only use geographical divisions but also consider sociodemographic and socioeconomic differences.

### Need to Improve the Health Literacy Level of Residents During Major Public Health Emergencies

In addition, we found that due to the impact of COVID-19, the health literacy levels of all age groups declined. In this epidemic, the Chinese government has taken measures to try to conduct home quarantine throughout the nation, which has had a huge impact on people's lives and behavior. Poor population health literacy is an underestimated public health problem worldwide, and the New Coronary Pneumonia Information Center emphasized this issue (25). In the crisis of the COVID-19, the health literacy of individuals, communities, and populations (26) were very important. On the one hand, the internet has made information easier to access (27), but on the other hand, it also contains a lot of false information (28) about health, which makes enhancing health literacy difficult. The epidemic has expedited the rapid spread of false health information (26), making the popularization of health literacy more challenging. On the other hand, in traditional health communication, it has been emphasized that the public should obtain health knowledge only from official channels and medical professionals. However, compared with common existing infectious diseases, the understanding of COVID-19 in the scientific community is also undergoing a process of continuous learning and updating, so there are often conflicts of opinions in the scientific field or differences before and after official releases. The instability of the authority of health information sources has affected the basic knowledge and concepts of residents of all ages.

Health literacy is equally important for the prevention of infectious and non-communicable diseases. Promoting the level of health literacy is necessary for navigating information, identifying false and misinformation, and making decisions based on reliable and credible information (13, 29). Therefore, further research is needed on how to overcome the huge influences of major public health emergencies such as COVID-19 on the health literacy of residents. In the NPC and CP PCC this year, China will significantly enhance its ability to respond to public health emergencies as a long-term goal in 2035.

### Pay Attention to the Prevention and Treatment of Chronic Diseases With Health Quality in Public Health Emergencies

To explore the abnormal phenomenon that the health literacy levels of residents of different age groups did not rise but instead fell after the outbreak of COVID-19, we analyzed the health literacy levels from various dimensions. Our results showed that in terms of the health literacy levels of chronic disease prevention and treatment, the health literacy levels of residents of different age groups showed a significant downward trend.

In our country, chronic diseases have become the main cause of death among residents (30), and their prevalence is increasing over time. Researchers have pointed out that age and the number of chronic diseases are high predictors of low health literacy (31), and treatment compliance and medication compliance are key factors in chronic disease management and treatment (32, 33). Therefore, health literacy plays an extremely important role in the treatment and management of chronic diseases. The data presented in the current article showed that the prevention and treatment of chronic diseases was the main reason for the decline in the health literacy level of residents in 2020. The epidemic interrupted chronic medical services and the supplies of medications for chronic diseases. These factors have had an impact on the health literacy of residents of all ages in terms of chronic disease prevention.

### Health Literacy of Different Dimensions of the Elderly Group Needs to Pay Special Attention Compared to Other Age Groups

Our results showed that the health literacy of the elderly in all dimensions was the lowest among all age groups. As China's aging degree intensified and the urbanization rate continued to increase, the health of the elderly was threatened. The current health literacy of the elderly in my country was generally low (34), but it was of great significance to improve the health literacy level of the elderly. Health literacy can independently predict the mortality of the elderly (35). Improving health literacy can improve the medication compliance which was a key factor in the treatment of chronic diseases in the elderly of elderly people with chronic diseases (36). The results of this article showed that the health literacy level of the elderly needed to be improved in the following four dimensions compared with the whole age: Basic Knowledge Concepts, Scientific Health Concepts, Safety and First Aid, and Basic Medical Care. For example, interventions aimed at health education and health promotion should be adopted to improve the health literacy of the elderly in the context of urbanization, especially those with lower socioeconomic status (37). Second, the possibility of receiving regular health checkups and reporting good self-assessed health conditions was significantly higher. Sufficient health information may be obtained from multiple sources (38). Therefore, according to the dimensions of health literacy, a variety of methods can be used to improve the health literacy level of the elderly in a targeted manner.

## Limitations

First, the main limitation of the study regards its cross-sectional nature, making it difficult to make causative inferences. Second, this study did not measure the health literacy levels of residents under 15 and over 69, so the results cannot explain the difference in health literacy between children and the elderly in Chongqing. Thirdly, because some of the data information was not detailed enough, we were unable to conduct an in-depth analysis of the reasons for the abnormal decline in residents' health literacy levels in 2020. In addition, the total sample size surveyed in 2020 was smaller than that in 2019 due to COVID-19 precautions.

## Conclusion

This study showed that there were significant differences in the comprehensive health literacy levels of residents of different age groups in Chongqing, and their risk factors were also distinct. Among the sociodemographic and economic factors, in order to solve the problem of urban and rural health quality gap, we should not only use the geographical divisions but also consider the differences between social population and social economy. We suggest that targeted interventions related to health literacy can be implemented for people of various age groups. Due to the COVID-19 outbreak, this study used the investigative year as a variable. The level of health literacy in some dimensions was significantly reduced in 2020, especially in terms of Basic Knowledge and Concepts and Chronic Diseases prevention. It is recommended that the health literacy education of residents should be strengthened during major public health emergencies. In particular, attention should be paid to the improvement of the health literacy of the elderly in the four dimensions of Scientific Health Concepts, Safety and First Aid, and Basic Medical Care. The above findings will provide more effective ways and ideas to improve the health literacy of residents of different ages.

## Data Availability Statement

The datasets for this article are not publicly available because: we cooperate with the data supplier (Chongqing Municipal Health Education Institute) involved confidentiality agreement, the original data can not be disclosed. Requests to access the datasets should be directed to Weiwei Liu, lww102551@cqmu.edu.cn.

## Ethics Statement

The studies involving human participants were reviewed and approved by the study was approved by the Ethics Committee of Chongqing Collaborative Innovation Center for Functional Food Approval (202009201H). Written informed consent to participate in this study was provided by the participants' legal guardian/next of kin.

## Author Contributions

PY and YO acquired and interpreted the data and drafted the manuscript. HY analyzed and interpreted the data. XP has made substantial contributions to conception and design. JL and YW revised the manuscript. FT and XZ acquired of data. WL involved in analysis and interpretation of data, revising the manuscript, and given final approval of the version to be published. All authors contributed to the article and approved the submitted version.

## Conflict of Interest

The authors declare that the research was conducted in the absence of any commercial or financial relationships that could be construed as a potential conflict of interest.

## Publisher's Note

All claims expressed in this article are solely those of the authors and do not necessarily represent those of their affiliated organizations, or those of the publisher, the editors and the reviewers. Any product that may be evaluated in this article, or claim that may be made by its manufacturer, is not guaranteed or endorsed by the publisher.
